# Endolysins: a new antimicrobial agent against antimicrobial resistance. Strategies and opportunities in overcoming the challenges of endolysins against Gram-negative bacteria

**DOI:** 10.3389/fphar.2024.1385261

**Published:** 2024-05-20

**Authors:** Fazal Mehmood Khan, Fazal Rasheed, Yunlan Yang, Bin Liu, Rui Zhang

**Affiliations:** ^1^ College of Civil and Transportation Engineering, Shenzhen University, Shenzhen, China; ^2^ Institute for Advanced Study, Shenzhen University, Shenzhen, China; ^3^ Institute of Microscale Optoelectronics, Shenzhen University, Shenzhen, China; ^4^ Southern Marine Science and Engineering Guangdong Laboratory, Zhuhai, China

**Keywords:** antimicrobial resistance, antimicrobial agent, *A. baumannii*, Gram-negative bacteria, endolysin

## Abstract

Antibiotic-resistant bacteria are rapidly emerging, and the increasing prevalence of multidrug-resistant (MDR) *Acinetobacter baumannii* poses a severe threat to humans and healthcare organizations, due to the lack of innovative antibacterial drugs. Endolysins, which are peptidoglycan hydrolases encoded by a bacteriophage, are a promising new family of antimicrobials. Endolysins have been demonstrated as an effective therapeutic agent against bacterial infections of *A. baumannii* and many other Gram-positive and Gram-negative bacteria. Endolysin research has progressed from basic *in vitro* characterization to sophisticated protein engineering methodologies, including advanced preclinical and clinical testing. Endolysin are therapeutic agent that shows antimicrobial properties against bacterial infections caused by drug-resistant Gram-negative bacteria, there are still barriers to their implementation in clinical settings, such as safety concerns with outer membrane permeabilizers (OMP) use, low efficiency against stationary phase bacteria, and stability issues. The application of protein engineering and formulation techniques to improve enzyme stability, as well as combination therapy with other types of antibacterial drugs to optimize their medicinal value, have been reviewed as well. In this review, we summarize the clinical development of endolysin and its challenges and approaches for bringing endolysin therapies to the clinic. This review also discusses the different applications of endolysins.

## 1 Antimicrobial resistance

The discovery of antibiotics transformed medicine, greatly increasing the quality of life, extending human lifespans, and enabling numerous complicated medical procedures and medicines, all while maintaining healthy lives and boosting public health. Unfortunately, genetically encoded antimicrobial resistance (AMR) has increased as discoveries have reduced, particularly in the early part of this century. Nonetheless, there have always been infectious factors when antibiotics have failed, and these keep outnumbering antibiotic resistance as a cause of morbidity and mortality (de la Fuente-Nunez et al., 2023). The resistance to antibiotics is a result of bacterial evolution, which protects bacteria from drugs that are harmful to their survival. It is a subset of AMR, a broader term for the evolution of resistance to naturally occurring agents or targeted medications in bacteria. AMR is linked to antibiotic use and becomes worse by antibiotic misuse and overuse in the medical sector. Increased resistance in pathogenic bacteria poses several critical public health problems, including severe and protracted illness, more hospitalizations and complications, and higher rates, all of which result in a significant cost burden (Behling et al., 2023). In recent decades, worldwide use of veterinary antibiotics has decreased, although worldwide clinical use of antibiotics remains high, and overuse exists in low- and middle-income nations. Although the majority of countries have implemented national action plans (NAPs) to actively address antibiotic resistance, actual AMR measures range significantly (Ding et al., 2023). In 2015, the 68th World Health Organization (WHO) assembly passed the global action plan on AMR to support action against resistance to antibiotics, and as a result, several nations and regions have implemented AMR mitigation strategies. The library of AMR NAPs was evaluated on 31 January 2023, and 134 countries (of which 40 were written in a language other than English) had officially approved NAPs, which includes 69% of the world’s 194 countries (Ding et al., 2023). As a result of antibiotic overuse and misuse, an increasing number of resistant bacteria have been isolated from the healthcare system and the environment. The rapid genetic material and resistance genes exchange among bacterial strains encourages the spread of AMR (Loh et al., 2021). Antibiotic resistance has been a worldwide challenge that could make a massive impact on both healthcare systems and the economic system. To fill the significant gap left by a high rate of resistance development and an inadequately filled antibiotic development pipeline, the WHO has called for the urgent development of novel antibacterial agents (Abdelkader et al., 2022). A novel alternative antimicrobial agent must be developed immediately to combat the severe threat of MDR bacterial infections due to the decline in antibiotic efficacy and the lack of novel antibiotics, pushing us closer to a post-antibiotic era (Chu et al., 2022). The bacteria that are part of ESKAPE (*Enterococcus faecium, Staphylococcus aureus, Kleibsiella pneumoniae, A. baumannii, P. aeruginosa* and *Enterobacter species*) are important drug-resistant pathogens. Among these, *Acinetobacter baumannii* is regarded as a global problem because of its potential to gain antibiotic resistance at a staggering rate (Rai and Kumar, 2022). *A. baumannii* is a significant nosocomial and opportunistic Gram-negative bacterium, causing infections worldwide, especially in intensive care units (ICU). It can cause numerous hospital- and community-acquired infections, such as ventilator-associated pneumonia, bacteremia, wound infections, and meningitis. It is clinically significant because of its capacity to survive for long periods on the substrate surface and its resistance to multiple drugs, making it the most prevalent and high alert pathogen on the WHO list of critical pathogens. This bacterium has been recognized to flout available therapeutic options. Alternative approaches to medicating *A. baumannii* infections are vitally important (Oyejobi et al., 2022a). The WHO has classified carbapenem-resistant *A. baumannii* (CRAB) as an urgently needed pathogen with limited therapy choices, thus there is an urgent need for research and development of new antibiotics as well as various alternative treatment strategies (Jansen et al., 2018). AMR highlighted that the present therapeutic pipeline is insufficient to address the AMR issue, particularly for three Gram-negative carbapenem-resistant, important priority pathogens: *Pseudomonas aeruginosa, A. baumannii,* and Enterobacteriaceae. Despite an increase in the number of antibiotics targeting Gram-negative infections in the clinical pipeline, most of them are variants of existing ones, serving as a temporary remedy because resistance to these evolved medications is likely to emerge quickly. Out of forty-two novel drugs for treatment that target priority pathogens, just two drugs from the same class (siderophore-cephalosporins) target the three Gram-negative critical priority pathogens (Gutiérrez and Briers, 2021). With a novel mode of action, bacteriophage-encoded endolysin has developed into the most effective class of bactericidal biological agents currently undergoing clinical testing (WHO (Organization, 2019). Endolysin were initially denied from use against Gram-negative pathogens due to their impermeable outer membrane, but they are now being developed as effective therapeutic agent against these critical priority pathogens. In principle, the three routes of investigation have been recently explored and advanced to varying degrees, including the use of endolysin that possess intrinsic activity due to a positively charged C-terminus that destabilizes the outer membrane, the use of physical or chemical methods to disrupt the outer membrane integrity, and protein engineering to provide the endolysin with the tools it needs to overcome the outer membrane (Gutiérrez and Briers, 2021). In this review article, we have specifically addressed the use of endolysin against *A. baumannii* and we have addressed and presented *A. baumannii* as a case study model of the use of endolysin as a new therapeutic agent. We have also highlighted the issues and barriers of transforming endolysin from the lab to the clinic and the strategies to overcome these issues of endolysin to be used as a novel therapeutic drug.

### 1.1 Endolysins’ structure and enzymatic activity

Endolysins are proteins that are expressed late in the bacteriophage virus’s lytic cycle. After the bacteriophage completes its lytic cycle within the host, endolysins break down the host’s cell wall by splitting the host’s peptidoglycan to release offspring virions (Abdelrahman et al., 2021b). Gram-positive bacteriophage endolysins have a modular structure, with EADs at the N-terminus and CBDs attached at the C-terminus via a linker. Endolysins have enzymatic hydrolysis and substrate identification capabilities due to the presence of EADs and CBDs. Typically, modular endolysins have one or two EADs at the N-terminal and CBD at the C-terminal connected by a flexible region known as the linker (Gondil et al., 2021b). The N-terminal EAD of modular endolysins cleaves certain peptidoglycan bonds in the host bacterium’s murein layer, while the C-terminal CBD identifies and connects to numerous epitopes in the cell wall for optimal binding of the EAD’s catalytic actions (Wang et al., 2023). The bacteriophage-derived endolysins of Gram-negative hosts can be structured in different kinds of ways, although most of them have a basic globular EAD domain without a CBD. The latest study also discovered Gram-negative endolysins with globular structures, one or two CBDs at the N-terminus, and the EAD module at the end of the C-terminal region (Briers et al., 2007). Endolysins are categorized based on their cleavage site. These enzymes include lysozymes (N-acetylmuramidases), glycosidases (N-acetyl--d-glucosamidases), N-acetylmuramoyl-l-alanine amidases, and L-alanoyl-d-glutamate endopeptidases (Khan et al., 2023b). Endolysins, which are composed of one of these four N-terminals connected to a distinct cell wall-binding domain, also be known as bacteriophage-encoded cell wall hydrolases (Murray et al., 2021).

Glycosidases: The polymeric structures of N-acetylmuramic acids (MurNAc) and N-acetylglucosamines (GlcNAc) are linked by −1,4 glycosidic linkages that are split by glycosidases. N-acetyl--D-muramidase’s split bonds within GlcNAc and MurNAc residues. N-acetyl--D-glucosidases split bonds within GlcNAc and MurNAc residues (Matamp and Bhat, 2019). Like the other two glycosidases, transglycosylases cleave −1,4 bonds within GlcNAc and MurNAc, but they additionally play a role in an intramolecular mechanism that results in the production of a 1,6-anhydro ring at the MurNAc residue (Rodríguez-Rubio et al., 2016a). Lysozymes, also known as N-acetylmuramidases, eradicate bacteria by specialized hydrolysis. Glycosidases, or N-acetyl-β-d-glucosamidases, control the hydrolysis of the glycosidic link. Glycosidic linkages β-1,4 bind the NAG (N-acetylglucosamine) and NAM (N-acetylmuramic acid) monomers of peptidoglycan polymers together. Lysozyme acts to break the structural integrity of the peptidoglycan cell wall by hydrolyzing these connections. This results in an imbalance in the turgor pressure, which kills the bacterial cell (Ragland and Criss, 2017).

Amidases: The mechanism by which N-acetylmuramoyl-L-alanine amidases, also referred to as peptidoglycan amidases act is to break the amide bond that separates the glycan strand from the stem peptide that is located between the L-alanine and N-acetylmuramic acid residues (Abdelrahman et al., 2021b).

Endopeptidases: Enzymes called endopeptidases dissolve the bonds that hold two amino acids together in the stem peptide. Both L-alanoyl-d-glutamate endopeptidases and interpeptide bridge-specific endopeptidases target the peptide that makes up the L-alanoyl-d-glutamate link. Between stem peptides or inside an interpeptide bridge, bonds can break (Rodríguez-Rubio et al., 2016a).

### 1.2 *A. baumannii* resistance and a lack of therapeutic alternatives

Treatment of *A. baumannii* infections is complex due to its high capacity for acquiring resistance genes and its natural resistance to a wide range of antibiotics (Poirel et al., 2011). *Acinetobacter baumannii* infections are frequently treated with colistin as a last-line antibiotic. Still, an increasing number of reports on colistin-resistant strains indicate that the era of antibiotics is challenging (Lim et al., 2019). Carbapenems were once the primary therapy for MDR *A. baumannii* infections, but their use has resulted in an increase in carbapenem resistance in recent years (Nordmann and Poirel, 2019). Polymyxins are currently routinely used as the antibiotics of choice for treating MDR *A. baumannii* infections, despite initially being prohibited due to systemic toxicities (nephrotoxicity and neurotoxicity) (Piperaki et al., 2019). Extensive drug-resistant (XDR) *A. baumannii* is classified as an isolate resistant to three or more of the antibiotic classes (penicillin’s and cephalosporins, which include inhibitor combinations, fluoroquinolones, and aminoglycosides, with carbapenem resistance in the majority of cases). In contrast, pan-drug-resistant (PDR) *A. baumannii* is an XDR isolate resistant to polymyxins and tigecycline (Mulani et al., 2019). Antibiotic resistance mechanisms fall into three categories. First, resistance can be developed by decreasing membrane permeability or increasing antibiotic efflux, thereby blocking access to the target. Secondly, bacteria can safeguard the antibiotic receptor through genetic mutation or post-translational modification. Finally, antibiotics can be immediately inactivated through hydrolysis or alteration (Blair et al., 2015). One of *Acinetobacter’s* most powerful weapons is its remarkable genetic adaptation, which allows for rapid genetic changes and rearrangements, as well as the incorporation of foreign determinants carried by mobile genetic components. Insertion sequences are regarded as one of the primary mechanisms shaping bacterial genomes and, ultimately, evolution (Vrancianu et al., 2020). Furthermore, *A. baumannii* can form biofilms, extending its ability to survive on healthcare equipment, such as ventilators in intensive care units (ICUs) (Pakharukova et al., 2018). However, the link between biofilm development and antibiotic resistance is currently unclear (Yang et al., 2019). Many therapeutic options have been failed by *A. baumannii*, making treatment of these infections difficult and, in some circumstances, impossible. Given the scarcity of treatment alternatives, there is a need for an innovative strategy and rethinking of remedies for combating this bacterium (Oyejobi et al., 2022b).

### 1.3 Endolysins are a new class of therapeutic products against *A. baumannii* infections

The emergence of antibiotic-resistant *A. baumannii* necessitates the development of alternative treatment options, with many global pharmaceutical industry players strategically choosing to discontinue or outsource novel antibiotic discovery programs. One of the most promising methods is endolysin therapy, which uses endolysin to combat bacterial pathogens (Khan et al., 2021). Endolysins are peptidoglycan hydrolytic enzymes encoded by bacteriophages that have great potential as a new class of antimicrobial therapeutic agents for multidrug-resistant bacteria (Gondil et al., 2021a). Due to the widespread emergence of antibiotic resistance, endolysins have drawn more attention as an alternative therapy. There is strong evidence that endolysins can externally degrade peptidoglycan without the aid of a bacteriophage. Their incorporation into therapeutic approaches has consequently created new opportunities for treating bacterial infections in the human, veterinary, agricultural, and biotechnology fields (Abdelrahman et al., 2021a). Endolysins are a desirable alternative due to their lytic potential against various bacterial species that cause human infections. Endolysins are the most successful alternative therapy for treating and curing MDR bacteria. According to reports numerous endolysins have been used successfully to treat bacteria resistant to antibiotics. The antibacterial treatment through bacteriophage-derived endolysin is tested in animal experiment models. Due to their extraordinary therapeutic potential against multidrug-resistant bacteria, endolysin has attracted much attention in recent years (Mehmood Khan et al., 2023). Endolysin are bacteriophage-derived proteins which have high specificity for bacteria, they have no detrimental effects on human or animal cells. Endolysin lacks many features that make antibiotics superior to them, such as no resistance and high specificity (Abdelrahman et al., 2021a; Gondil et al., 2021b). Most bacteriophage endolysins indicate near-species specificity, which is thought to be one of their most beneficial features in this age of broad-range resistance to antibiotics as it avoids selective pressure on naturally occurring beneficial microbiota. Furthermore, resistance to endolysins is improbable for a variety of reasons, Endolysins have evolved to attach to and break highly conserved components in the cell wall of the pathogenic bacteria, secondly, as the bacteriophage and host bacteria coevolution occurs so there are fewer chances of resistance development to endolysin. Furthermore, because endolysins are applied externally and act on the cell wall rather than entering the bacterial cell, they avoid the majority of possible resistance mechanisms (e.g., active efflux from the cell or decreased membrane permeability) that contribute to resistance to most conventional antibiotics. Several endolysins include two catalytic domains that hydrolyze distinct bonds in the peptidoglycan, which is thought to limit the likelihood of resistance formation (Haddad Kashani et al., 2018).

Endolysin LysAB1245 showed broad lytic range efficacy against *A. baumannii* isolates from various capsular variants. Furthermore, LysAB1245 exhibited quick antibacterial activity and stability under different ranges of pH and temperature conditions. This study tested the possibility of LysAB1245 as a novel therapeutic agent for the treatment of MDR A. baumannii bacterial infections (Soontarach et al., 2024). Table 1 shows the summary of the therapeutic potentials of bacteriophage derived endolysins against *A. baumannii*.

**TABLE 1:** Endolysin against *A. baumannii*.

Endolysin	Antibacterial activity spectrum	Outcome	References
AbLys2	*A. baumannii*	• AbLys2 has a strong affinity for *A. baumannii*	Premetis et al. (2023a)
		• AbLys2 has effective lytic and antibacterial activity	
lysAB-vT2	*A. baumannii*	• lysAB-vT2 disrupts the mature bacterial biofilm	Sitthisak et al. (2023a)
LysAB54	*A. baumannii*	• LysAB54 is encoded by a novel *A. baumannii* bacteriophage p54, which showed a broad range of antibacterial activity against Gram-negative bacteria	Khan et al. (2021)
		• Shows a 4-log reduction activity against *A. baumannii* without the use of OMPs	
		• LysAB54 exhibited antibacterial activity against both the logarithmic and stationary phases of *A. baumannii*	
LysP53	*A. baumannii*	• LysP53 is active against a wide range of antibiotic-resistant Gram-negative bacteria, including *A. baumannii*	Li et al. (2021)
		• LysP53, 100 μg/mL showed a 5-log reduction of *A. baumannii* viable number	
LysSS	*A. baumannii*	• LysSS inhibits the growth of *A. baumannii* and *P. aeruginosa*	Kim et al. (2020)
	*P. aeruginosa*	• A549 human lung cells are not cytotoxic to LysSS.	
		• An intraperitoneal administration of LysSS protects mice infected with *A. baumannii* from systemic infection	
LysAB2P3	*A. baumannii*	• LysAB2 P3 reduced the bacterial burden by 13 times in ascites and 27 times in blood in a mouse intraperitoneal infection model	Peng et al. (2017)
		• LysAB2 P3 prevented deadly bacteremia in 60% of mice with severe *A. baumannii* infections	
LysAB2-KWK	*A. baumannii*	• LysAB2-KWK showed disruption of bacterial biofilms	Chen et al. (2021)
		• Showed activity against the stationary phase of *A. baumannii*	
		• LysAB2-KWK protects the larvae of *Galleria mellonella* from infection with *A. baumannii*	
		• It exhibits a long storage life, moderate serum tolerance, and a broad antibacterial spectrum against Gram-negative bacteria	
Ablysin	*A. baumannii*	• Strong antimicrobial activity in the absence of an outer membrane permeabilizer	Vasina et al. (2021)
		• Lysis activity against multi-drug resistant clinical strains of *A. baumannii*	
		• Antibacterial and biofilm-removing activities against *A. baumannii*	
LysAB3 and LysAB4	*A. baumannii*	• LysAB3 and LysAB4 100 μg/mL disrupt the *A. baumannii* cell shape	Lai et al. (2013)
Abtn-4	*A. baumannii*	• Showed a 3-log reduction against *A. baumannii*	Yuan et al. (2021)
		• Anti-biofilmactivity against bacterial biofilm	
		• Antimicrobial activity against phage-resistant bacterial mutants	
DS-PA90	*A. baumannii*	• It can completely lysis the *A. baumannii* at 0.25 µM concentration	Lim et al. (2022)
Artilysin Art-175	*A. baumannii*	• High antibacterial activity against multidrug-resistant *A. baumannii* stationary-phase cells	Defraine et al. (2016)
		• It can eliminate huge inoculum (10^8^ CFU/mL)	
LysMK34	*A. baumannii*	• LysMK34 exhibited intrinsic antibacterial activity against *A. baumannii* strains up to 4.8 log units in the presence of turgor pressure	Abdelkader et al. (2020)
		• Tolerate temperatures up to 55°C, as well as a wide pH activity range (4–10)	
LysABP-01	*A. baumannii*	• LysABP-01 can denature the rough cell wall of the *A. baumannii* strain	Thummeepak et al. (2016a)
		• It showed synergist activity with colistin	
AbLys1	*A. baumannii*	• AbLys1 have high specificity for *A. baumannii*	Premetis et al. (2023b)
		• It is most active at pH values of 7–8	
LysAB2	*A. baumannii*	• LysAB2 lysis the *A. baumannii* peptidoglycan	Lai et al. (2011a)
PlyF307	*A. baumannii*	• It can efficiently kill more than >5-log-unit reduction of *A. baumannii* clinical strains	Lood et al. (2015)
		• PlyF307 treatment disrupted the biofilm of *A. baumannii*	
		• PlyF307 saved mice from potentially deadly *A. baumannii* bacteremia	
P307 and P307Q-8C	*A. baumannii*	• P307 and P307SQ-8C both exhibited strong *in vitro* efficacy against biofilms of *A. baumannii*	Thandar et al. (2016)
		• P307SQ-8C diminished the bacterial burden in a mouse model of *A. baumannii* superficial infection by 2 logs	
PlyAB1	*A. baumannii*	• PlyAB1 has lytic activity against the *A. baumannii* AB1 strain	Huang et al. (2014)
		• It did lysis of 48 *A. baumannii* isolates that are resistant to drugs	
Ply6A3	*A. baumannii*	• The intraperitoneal injections of endolysin Ply6A3 rescue deadly *A. baumannii* sepsis mice *in vivo*	Wu et al. (2019)

### 1.4 Evaluation of endolysin against *A. baumannii*

Establishing the *in vivo* safety and efficacy of phage-encoded endolysin as medicinal products is an essential step in their development (Lai et al., 2020a). Endolysin can be administered by various methods, such as intravenously and intraperitoneal injections, topical treatments (creams, ointments, and gels), and trans nasal, vaginal, and oral delivery systems (Abdelrahman et al., 2021b). Endolysins, which target pathogens in various tissues and organs, are currently in clinical trials. Schmelcher et al. have briefly addressed the systemic use of endolysins to treat bacteria-related infections of the bloodstream, organs, and tissues (Schmelcher M. and Loessner M. J. J. C. O. I. B., 2021). In an *A. baumannii* mouse model of burn wound infection, a single dosage of 14 µg/mouse of endolysin LysP53 resulted in a 3-log reduction in bacterial load. The findings suggested that LysP53 can be a promising choice for the treatment of topical infections caused by *A. baumannii* and other Gram-negative bacteria (Li et al., 2021). ElyA1 was used to treat skin infections of *A. baumannii* in *Galleria mellonella*. ElyA1 showed a confirmed significant reduction in the number of bacteria (Blasco et al., 2020a). The P307_SQ-8C_ reduced the bacterial burden by two logs in a mice model of *A. baumannii* cutaneous infection in 2 h (Thandar et al., 2016). LysAB2 P3 reduced bacterial burden in ascites and blood by 13-fold and 27-fold, respectively, in a mouse intraperitoneal infection model 4 h after bacterial injection. Furthermore, LysAB2 P3 saved 60% of mice infected with *A. baumannii* from deadly bacteremia (Peng et al., 2017). Repeated exposure to endolysins LysAm24, LysAp22, LysECD7, and LysSi3 that target Gram-negative bacteria are tested to determine the development of resistance. These endolysins function well in animal models of wound and burn skin infections, have a broad spectrum of activity, and are active against both planktonic bacteria and bacterial biofilms. Regarding safety, these enzymes do not cause cytotoxicity, do not contribute to the development of resistance, and do not disrupt the natural intestinal microbiota *in vivo* (Vasina et al., 2021). Endolysins specifically prevent specific species or subspecies of pathogenic bacteria, without disrupting the surrounding normal microbiota (Yoong et al., 2004). The endolysin PlyV12 causes less disruption of the normal microbiota when used to treat antibiotic-resistant bacterial strains and they do not transfer resistance genes or bacterial toxins and do not eliminate the colonized beneficial normal flora of mucous membranes (Rahimzadeh et al., 2018).

### 1.5 Endolysin against *A. baumannii* biofilm

Due to several resistance mechanisms, including biofilm formation, *A. baumannii* can survive and spread in the hospital environment (Salmani et al., 2020). Endolysin chemotherapy has a significant antibacterial effect on biofilm-associated bacteria, which are difficult to eliminate due to their minimal metabolic activity (Chang et al., 2022). Endolysin Abtn-4 treatment inhibited *A. baumannii* biofilm growth by more than 30%, suggesting that endolysin is a possibly effective antibacterial agent in regulating biofilm development (Yuan et al., 2021). The pre-treatment of catheters with endolysin PlyF307 decreased *A. baumannii* biofilm on the surface by 1.6 log10, resulting in a substantial decrease in total biofilm growth (Lood et al., 2015a). In an additional *in vivo* study, Lood et al. investigated the efficiency of PlyF307 in eliminating biofilm from subcutaneously implanted catheters colonized with 2-day-old *A. baumannii* biofilms in the intestines of mice. Their findings revealed that mice with fatal *A. baumannii* bacteremia were saved by PlyF307 (Lood et al., 2015a). Endolysin anti-biofilm activity should be evaluated in future studies implementing more clinically relevant models such as multispecies biofilm matrices and flow cell-based biofilm models, as well as pre-treatment processes on diverse substrates and surfaces in hospitals (Chang et al., 2022). The endolysin LysAB1245 showed broad lytic spectrum efficacy against MDR *A. baumannii* isolates from various main capsular types. Furthermore, LysAB1245 has the potential for use with nosocomial MDR *A. baumannii* infections and can control the biofilms in the clinical healthcare environment (Soontarach et al., 2024).

### 1.6 Synergistic effects of endolysin and antibiotic against *A. baumannii*

The outer membrane of Gram-negative bacteria acts as a barrier for many endolysins, and very few endolysins with exogenous activity against Gram-negative bacteria have been observed. Endolysins can target Gram-negative bacteria if the outer membrane is first permeated with chemicals such as Ethylenediaminetetraacetic acid (EDTA), which destabilizes the lipopolysaccharides of the outer membrane; but the combined use of endolysin and EDTA is restricted to superficial treatment of localized infection (Briers et al., 2011; Thummeepak et al., 2016b). Several studies have tried enhancing the muralytic effect of endolysins by using them with different antibiotics to take on their combined synergistic effects (Thummeepak et al., 2016b; Letrado et al., 2018). Using endolysin and antibiotics together to target Gram-negative bacteria has shown some promising outcomes (Lai et al., 2020a). The synergistic effect of LysABP-01 endolysin with seven commonly prescribed antibiotics against the MDR strain of *A. baumannii* was assessed. LysABP-01 and colistin together had a synergistic effect *in vitro*, with a fractional inhibitory concentration index ranging from 0.156 to 0.188. The encouraging findings demonstrated that colistin and LysABP-01’s minimum inhibitory concentrations (MICs) were lowered by up to 8 and 32 fold, respectively (Thummeepak et al., 2016a). Colistin, a ‘last-resort’ antibiotic, is commonly used to treat infections caused by MDR Gram-negative bacteria. However, due to its nephrotoxicity and neurotoxicity, the dose of colistin is extremely restrictive (Nation and Li, 2009). As a result, combining colistin with lysins may help optimize the clinical use of colistin at a lower dose. Blasco et al. recently demonstrated the synergistic effects of the endolysin ElyA1 and colistin in targeting various MDR Gram-negative bacteria including *A. baumannii*, in both *in vitro* and *in vivo* conditions. When colistin was combined with ELyA1, the MIC of colistin was lowered by at least 4-fold for all tested *A. baumannii* strains (Blasco et al., 2020a). In both skin and lung infection models, the *in vivo* survival rate for *A. baumannii* infected *G. mellonella* and bacterial reduction of infected mice treated with ELyA1 and colistin combination was considerably greater than that of the colistin-alone. Colistin can operate as an OMPto break the integrity of the OM, allowing lysin access to the PG substrates. Simultaneously, peptidoglycan cleavage by endolysin may increase antibiotic absorption and hence promote antibiotic action (Gerstmans et al., 2018a). These positive results show that combining endolysin and antibiotics has the potential to resensitize bacterial pathogens with drug resistance to antibiotics. And to slow the spread of antibiotic resistance by using fewer antibiotic treatments of low dosages (Lai et al., 2020a). Combined use of endolysin LysAB-vT2-fusion with colistin, polymyxin B, or copper showed synergistic against *A. baumannii*, which may be used for the control of *A. baumannii* infection (Sitthisak et al., 2023b).

## 2 Clinical development of endolysin

Protein prescription drugs are a rapidly growing sector of the pharmaceutical industry. According to THPdb, a curated database of FDA-approved therapeutic proteins and peptides, there are 239 approved proteins and peptides, as well as 380 recognized variants of these proteins/peptides (Usmani et al., 2017; Wishart et al., 2018). Protein enzymes have significant advantages as medications because of their high selectivity, proteinaceous composition, which eliminates chemical toxicity, and tremendous potential for modification and consequent development (Labrou, 2019). Endolysins have been recognized by the WHO as a novel, non-traditional antimicrobial (WHO (Organization, 2022). Several biotechnology and pharmaceutical companies have been performing human clinical trials utilizing endolysins. ContraFect, a biotech company located at Rockefeller University, obtained ownership rights to nine phage-derived endolysin. This company particularly focuses on endolysin therapy for bacterial infectious diseases. ContraFect conducts a phase III clinical medicine trial on endolysin (identifier#: NCT04160468). At the same time, iNtRON Biopharma completed a phase III clinical trial of N-Rephasin Sal200 in 2021. (clinicalTrials.gov, accessed on 21 June 2022, identifier#: NCT03089697). In February 2020, the FDA recognized endolysin innovations as antibacterial biological drugs by recognizing the ContraFect endolysin in the phase III study as “Breakthrough Therapy.” Pre-clinical development of additional endolysins is now underway in both industries and universities (Harhala et al., 2022). Exebacase “Endolysin CF-301” is a recombinant endolysin developed by ContraFects for use against a variety of *Streptococcus* and *Staphylococcus* species in the treatment of infective endocarditis in humans. This endolysin was the first of its class to enter human clinical trials in the United States. In phase II clinical studies, endolysin enhanced the recovery rate of methicilin-resistant *S. aureus* (MRSA) induced infectious endocarditis by 42.8% when used in combination with regular antibiotic treatment (Watson et al., 2019). Another phage-derived endolysin that has shown clinical efficacy is SAL200. SAL200, also known as N-Rephasin^®^ SAL200, was found to be an acceptable medication for intravenous administration in this trial. Thirty-four healthy male volunteers received SAL200 at varying doses, There were no significant adverse effects noted during the endolysin pharmacokinetics study in the human body and its tolerance to intravenous administration (Jun et al., 2017).

## 3 Challenges of endolysin therapy transferring from the lab to the clinic

Totté et al. reported three clinical cases in which patients with persistent and recurring *S. aureus*-related skin infections were successfully treated with Staphefekt SA.100. Staphefekt SA.100 is a synthetic phage endolysin designed for topical skin application. Staphefekt is currently registered as a (class 1) medical device in Europe and is readily accessible over the counter as a cetomacrogol-based cream and gel (Totté et al., 2017). Although increased interest has been directed to investigating the potential of endolysin to target MDR Gram-negative bacteria, there are several challenges to its clinical application. These include questions about the safety of OMPs with endolysin, the application of outcomes from animal models of acute infection to clinical practice, and the effectiveness and stability of endolysin. Protein engineering and formulation design for effective distribution and stabilization of Gram-negative endolysin could be some options for advancing Gram-negative endolysin development for clinical testing before regulatory authorization (Lai et al., 2020a).

**FIGURE 1:**
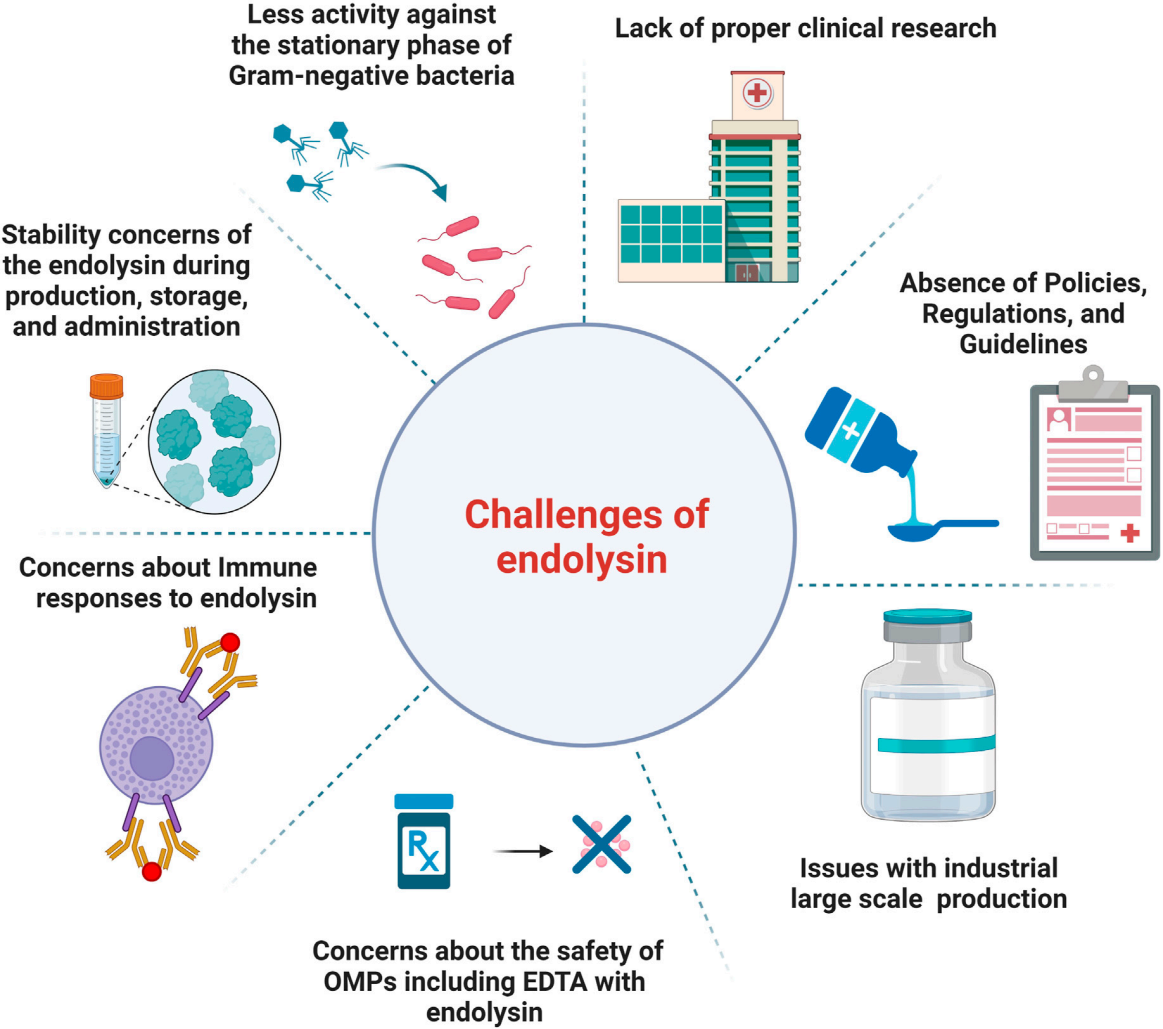
An illustration of the current challenges of endolysins use in the clinic and healthcare market.

Figure 1 describes the current challenges of endolysin use in the clinic and healthcare market.

### 3.1 Concerns about the safety of OMPs with endolysin

In Gram-negative bacteria, the outer membrane functions as a barrier to several endolysins.

Few endolysins with exogenous activity against Gram-negative bacteria have been reported and the majority are synthetically engineered endolysins (Lai et al., 2011b; Briers et al., 2014a; Briers et al., 2014b; Lim et al., 2014; Lood et al., 2015; Soontarach et al., 2024).

OMPs have the potential to significantly increase the antibacterial activity of endolysin while also broadening their host spectrum. However, the *in-vivo* study of the efficacy of endolysin and OMPs in combination is rarely described in the literature. The harmful effects of the most often used OMPs such as EDTA through various delivery routes, such as oral, intravenous, intraperitoneal, topical, and inhalation, have been investigated (Lanigan and Yamarik, 2002). EDTA is cytotoxic, slightly genotoxic, and has an anticoagulant effect. It has been observed that the low EDTA dose required to cause toxicity in rats was 750 mg/kg/day. As a result, the use of EDTA with endolysin should be restricted to topical applications because it cannot affect the skin and does not produce skin hypersensitivity (Lanigan and Yamarik, 2002). Citric acid is also used as an OMP, but despite having a much better safety profile than EDTA, more testing is required because citric acid also has hazardous consequences, When used in combination with endolysin, particularly for the inhalation route, as it has been linked to animal obstruction of the airway and cause reactions in humans coughing (Allott et al., 1980). With further work being done on combining endolysin with polycationic peptides to enable them to target Gram-negative bacteria, their therapeutic use should be carefully evaluated. This is because endolysin with OM-disrupting characteristics or those coupled with peptide OMPs may also interact with eukaryotic cell membranes, raising safety issues (Schmelcher et al., 2012).

### 3.2 Weak effectiveness against the stationary phase of Gram-negative bacteria

The log-growth phase bacteria are more susceptible to endolysin treatments than the stationary phase bacteria. Oliveira et al. presented that the lower bactericidal efficiency against the bacterial stationary cells is due to modifications to the structure in the outer membrane, such as LPS biosynthesis, which affects endolysin permeability that even in the presence of OMPs, or chemical modifications of the peptidoglycan compositions (Oliveira et al., 2014). These alterations could aid bacterial cells in coping with endolysins *via* acetylation, N-deacetylation, and amidation (Typas et al., 2012). A significant number of findings show anti-bacterial efficacy against the log-phase bacteria. To overcome this research gap, and to completely investigate the potency of endolysin against Gram-negative bacteria, further research is required to evaluate the antibacterial efficiency of endolysins against the stationary phase bacteria. Regarding the concern of the resistance of bacteria to endolysin; The resistance development of endolysin was evaluated in which bacterial strains were repeatedly challenged with subinhibitory doses of endolysins or artilysins failed to give rise to resistant mutants, although bacterial strains exposed to control antibiotics got resistance. The probability of developing resistance against endolysins and artilysins is thus regarded as minimal, especially when compared to standard antibiotics (Fischetti, 2005; Briers et al., 2014a).

### 3.3 Concerns about stability

The stability of the endolysin during production, storage, and administration is essential for its prospective use as an antibacterial agent. Several stability concerns of Gram-negative endolysins have been observed in preclinical studies and must be solved for further research and development (Lai et al., 2020a). The oral delivery route is the most widely used for gut-targeted medicines and is also the most well-liked by patients. Most medications available on the market are used orally and come in tablet or liquid form. This is not without its challenges, though. Because medications of this kind have to pass through the stomach and digestive tract, they are instantly exposed to a variety of enzymes, varying pH levels, and mechanical digestion. All of these may have an impact on the drug’s molecular integrity and systemic bioavailability (Murray et al., 2021). Endolysins are proteinaceous, therefore they can be swiftly damaged by these processes, rendering them ineffective. Stomach acid has the potential to damage the structure of several endolysins. Proteases such as trypsin, chymotrypsin, pepsin, and peptidase degrade proteins. Many endolysins contain cleavage sites that these enzymes can target (Bruno et al., 2013).

#### 3.3.1 Inactivation of endolysin in physiological conditions

Environmental conditions such as salts and proteins are well recognized to have a major impact on the activity of Gram-negative endolysins. Khan et al. reported that the *A. baumannii* endolysins LysAB54 entirely loses its antibacterial efficacy in the serum, limiting its use to topical infections such as burn wounds or joint infections. One possible explanation for the reduction of LysAB54 activity in the serum is endolysin ion exchange, which might potentially neutralize endolysin antibacterial activity (Khan et al., 2021). Endolysin inactivation in human serum could be associated with the conjugation and passivation of positively charged peptides on endolysin by negatively charged serum molecules. The neutralized cationic domain may result in the lack of intrinsic OM permeabilizing activity (Yang et al., 2015).

#### 3.3.2 Thermostability

Because of their proteinaceous composition, endolysins are temperature-sensitive. Temperature effects on endolysin activity have been extensively studied since protein thermostability can affect functionality and storage stability. Most endolysin perform best activities between 30°C and 40 °C, making them useful for clinical use (Lai et al., 2020b).

#### 3.3.3 Storage stability

Drugs with a moderately extended shelf life (12 months) under refrigeration (2°C–8°C) or at room temperature (20°C–25°C) are preferable for commercial viability. Products for freezer storage (−20°C ± 5°C) should have at least a 12-month shelf-life (Guideline, 2003). However, there is a lack of information regarding the Gram-negative endolysin storage stability, and what little that is available only covers short-term storage—from 1 week to six months—under various storage environments (Lai et al., 2020b).

### 3.4 Concerns about immune responses to endolysins

One of the limitations of endolysin is its short *in vivo* half-life, caused by the inflammatory response of cytokines and neutralizing antibodies. Endolysin elicits an immune system response when used repeatedly, and as a result of the immune response, it loses its enzyme-mediated lytic activity *in vivo* (Nau and Eiffert, 2002; Jado et al., 2003; Abdelrahman et al., 2021a). Raz et al. described a novel approach to discovering immune-derived engineered endolysins. The engineering of CBDs of different endolysins fusions to the Fc receptor of human IgG antibodies. The engineered lysin called Lysibodies binds to *S. aureus* cells and opsonizes them, causing immune complement system activation, which can cause phagocytosis and clearance of the bacteria. Through this approach, endolysin can be used to target an immune response toward pathogenic bacteria (Raz et al., 2018). A similar approach without using antibody fragments was described for a protein derived from the CBD of the endolysin PlyV12 (Yang et al., 2018). As some of the Gram-negative endolysin enter preclinical development, concerns about immunogenicity, toxicity from lipopolysaccharide release during the bactericidal process, and pharmacokinetic issues due to the complexity of the Gram-negative cell wall must be addressed (Ghose and Euler, 2020). We suggested a strategy to minimize the body’s immune responses to endolysin. Engineered endolysins should be designed with human immune cells. When such proteins are expressed, the immune cells will recognize them as their body proteins, so there will be fewer immune reactions to these foreign proteins and less chance of the clearance of such foreign proteins in the body.

### 3.5 Lack of proper clinical research

In February 2020, the FDA recognized endolysin innovations as antibacterial biological drugs in the phase iii study as “Breakthrough Therapy” (Harhala et al., 2022). However, the fundamental obstacle to the use of endolysins as a therapeutic agent is the lack of proper clinical research to provide an excellent scientific fundamental basis for approval. We suggested that additional research on endolysins affecting their safety in humans will be beneficial to get past the reservations point of view on endolysins’ usage and create the legal framework for the approval of endolysins. Preclinical trials of more endolysins are needed to bring endolysin into the medical market for treatment purposes.

### 3.6 Absence of policies, regulations, and guidelines

Numerous potential endolysin applications are currently being reported in medicine, but there are no published guidelines and regulations for endolysins. Early and effective communication with the relevant authorities is essential to establish endolysin regulatory pathways in their use as a medicine in the health sector and medicine industry. However, there are currently no clear legal principles or laws governing the use of endolysins.

### 3.7 Issues with large-scale industrial production

Endolysins have made great progress towards clinical application as novel antibacterial treatments. Another major problem that must be addressed is the large-scale industrial manufacture of endolysin. The present cost of endolysin production is projected to be expensive, which may be a major barrier to the use of endolysins as antibacterial replacements (Oliveira et al., 2012). The high cost arises mostly from the necessity for the development of recombination engineering, effective, and safe expression platforms for endolysin expression and purification. As a result, technical techniques to increase the solubility and expression levels of target endolysins, as well as cost-effective expression systems, are required to make endolysins commercially acceptable antibacterial agents worth investing in (Lee et al., 2023). When transferring the therapeutic endolysin expression and purification from the lab to the industrial scale, manufacturing costs and safety concerns must be addressed. This applies to the entire manufacturing process, from the selection of the expression host. From the beginning of protein expression until the last stages of processing, there are numerous chances to lower production costs and improve safety. Cell harvesting, cell lysis, and purification are examples of downstream processing procedures that are carried out once endolysins have been engineered and reassembled in a particular expression system. Toxic effects must be avoided, especially when it comes to protein expression in bacteria where endotoxin must be eliminated. Furthermore, early in the production process, choosing the best expression hosts—such as bacteria, yeast, or plants—is a crucial step toward achieving overexpression and cheap expression costs. Because they are inexpensive to produce, plants have been suggested as potential hosts for recombinant protein production. Furthermore, plant-produced proteins are likely safer than those made from bacteria or animal cells (Magnusdottir et al., 2013).

#### 3.7.1 High dose of endolysin needed for efficient killing of Gram-negative bacteria

The majority of Gram-negative bacteria require dosages of 100–500 μg/mL of endolysin to be eradicated, which is significantly higher than that of their Gram-positive counterparts, which commonly need doses of less than 10 μg/mL (Abouhmad et al., 2016; Gerstmans et al., 2018b). The development of commercially viable products might encounter production and formulation issues due to the high concentration of endolysin needed. Reducing the amount of endolysin dose by boosting the potency of Gram-negative endolysin is an efficient way to deal with this problem. Protein engineering could be able to perform this higher lytic activity, meaning that, in comparison to natural lysins, a lower concentration (<100 μg/mL) of modified endolysin is needed to exert effective bacterial killing. The logical design of engineered endolysin is crucial for successful modification to improve activity and stability. To attain this goal, knowledge of the endolysin tertiary or crystal structures will be required (Lai et al., 2020b).

### 3.8 Limited availability of *in vivo* data

So far, the effectiveness of Gram-negative endolysin *in vivo* has only been assessed using acute infection models. There are fewer documented chronic *in vivo* models for endolysin assessment, except for topical infection research investigations (Lai et al., 2020b). The current *in vivo* research on Gram-negative endolysin has been focused on assessing the rates of survival and/or reduction of bacterial burden to validate their effectiveness. Before continued research, issues with immunogenicity, pharmacokinetics, and the production of proinflammatory components during bacteriolysis must be resolved for the systemic use of Gram-negative endolysin. Many Gram-positive endolysins had problems being cleared by neutralizing antibodies because they were proteinaceous, which resulted in a relatively short half-life in vivo—between 4 and 40 min, depending on the type of endolysin (Gerstmans et al., 2018b).

## 4 Overcoming challenges of Gram-negative endolysins through genetic engineering

The majority of naturally occurring endolysins are lytic against Gram-positive bacteria. In Gram-negative bacteria, the outer membrane inhibits endolysin from accessing the target peptidoglycan. Various scientific and formulation solutions have been developed throughout the years for tackling the outer membrane barrier (De Maesschalck et al., 2020). Modular endolysins offer a unique opportunity for scientists to engineer proteins to change bacteriolytic activity, solubility, specificity, and various other physicochemical properties (Haddad Kashani et al., 2018). The shortening of a full-length enzyme, site-directed mutagenesis, or different fusions such as EADs with cell wall binding domain CBDs of distinct endolysin, virion-associated endolysin with CBD of a different endolysin, endolysin fusion with antimicrobial peptides are examples of engineering techniques (Love et al., 2018).

### 4.1 Formulation strategies

Formulation techniques include the use of different carrier systems. The use of OMP (chelators such as citric acid, lactic acid, malic acid, acetic acid, benzoic acid and EDTA in the formulation is useful in permeabilizing the outer membrane of Gram-negative bacteria to endolysins (Oliveira et al., 2016a). Using OMP is the simplest way to get over the outer membrane barrier. The most often utilized permeabilizer in this scenario is EDTA, which works by chelating the outer membrane stabilizing divalent cations (Kocot et al., 2023).

### 4.2 Endolysin encapsulation system

Despite their therapeutic effects, bacteriophages and endolysins face some practical challenges posed by the host system, such as low bioavailability, losing activity, non-targeted delivery, rapid elimination by the retinal endothelial system, and antibody-mediated deactivation. Against this context, there has been an increase of interest among scientists to investigate the possibilities of delivery systems for encapsulating bacteriophages and endolysins. In recent years, a multitude of bacteriophage and endolysin encapsulation strategies have been reported (Loh et al., 2021). Similarly, nanoencapsulation can help improve the therapeutic potential by not only shielding endolysin from degradation but also allowing for continuous release, potentially boosting stability, shelf life, and therapeutic efficacy (Gondil and Chhibber, 2021). Liposomes’ ability to penetrate bacterial outer membranes through membrane fusion is also a viable way for delivering endolysin to Gram-negative bacteria. The authors achieved successful lysis of both *Salmonella typhimurium* and *E. coli* with endolysin BSP16Lys-containing liposomes without the need for a membrane permeabilizer (Bai et al., 2019b). These examples highlight the need to utilize these existing strategies to improve the potential of endolysin as an effective and sustainable therapy.

### 4.3 Endolysin delivery using liposomes

It has been observed that endolysin encapsulated in liposomes enhances the efficacy of endolysins against Gram-negative bacteria (Bai et al., 2019a). The method of packing lytic proteins derived from bacteriophage in the structure of liposomes appears to be a promising weapon against Gram-negative bacteria. Like dendrimers, liposomes can also have a protective role that maintains the endolysin activity (Kocot et al., 2023).

### 4.4 Endolysin delivery using chitosan nanoparticles

Encapsulation in alginate-chitosan nanoparticles is another strategy for increasing the efficacy of endolysin, particularly for use in therapy (Gondil and Chhibber, 2021). Chitosan-based formulations provide benefits over alternative delivery systems in that they boost therapeutic agent bioavailability while also properly removing from the host after the release of the agent (Gondil and Chhibber, 2021).

### 4.5 Breaching the barrier of the outer membrane of Gram-negative bacteria

Because the outer membrane (OM) of Gram-negative bacteria prevents externally applied endolysins from accessing peptidoglycan, controlling the growth of these Gram-negative pathogenic bacteria is difficult (Sisson et al., 2024b).

#### 4.5.1 Synergy through permeabilizing molecules

Chemical permeabilizers are a useful tool for facilitating endolysins’ entry into the periplasm. Divalent metal ion (Mg2+ and Ca2+) chemical chelate stabilizes the lipopolysaccharide (LPS) layer, degrades and permeabilizes the OM, and makes the peptidoglycan accessible to endolysins (Oliveira et al., 2016b). Citric and malic acids are examples of organic acids that permeabilize the OM and improve endolysins’ ability to kill Gram-negative bacteria (Sisson et al., 2024a). Endolysins work in combination with additional compounds that disrupt the OM. Oregano and thyme essential oils, carvacrol, have been shown to enhance endolysin activity against Gram-negative bacteria and could cause the breakdown of lipopolysaccharides. Moreover, colistin, polymyxin B, and ε-poly-L-lysine, which are cationic chemicals, work in combination with endolysins by either dissolving or competitively displacing OM cations. In summary, many chemicals allow endolysins to access peptidoglycan and promote cell lysis by damaging or permeabilizing the OM (Blasco et al., 2020b; Ning et al., 2021; Hong and Myung, 2022).

#### 4.5.2 Synergy through bacterial receptors

An alternate method for increasing endolysin activity is to enhance its attachment to and permeation of the OM. Adding endolysins to proteins allows them to attach to bacterial cell surface receptors. For example, ‘Lysocins’ are endolysin-bacteriocin fusions (Martínez et al., 2016; Heselpoth et al., 2019). Some bacteriocins produce pores in a membrane by binding to OM phospholipids and specific surface components, such as receptors and transporters, allowing the endolysin to pass through to the peptidoglycan (Martínez et al., 2016). Innolysins include the attachment of endolysin EADs to phage receptor-binding proteins (RBPs) and provide a second way of breaking through the OM (Zampara et al., 2020).

### 4.6 Engineering of endolysin with antimicrobial peptide

Some endolysins contain peptides, typically at their C-terminus, that have a strong antibacterial activity that is not dependent on peptidoglycan breakdown. These antimicrobial peptides (AMPs) are responsible for some endolysins natural capacity to penetrate the outer membrane of Gram-negative bacteria (Szadkowska et al., 2022; Kocot et al., 2023). Predicting AMP-like regions is a good technique for designing endolysin with an intrinsic antibacterial activity that can serve as a framework for further engineering (Kocot et al., 2023).

However, Gram-negative bacteria are difficult to regulate due to the presence of an outer membrane that protects the peptidoglycan layer from enzymatic breakdown. To surpass this threshold, the fusion of endolysin with a sensitizer peptide is known to extend efficacy by disrupting the outer membrane of Gram-negative bacteria (Son et al., 2023). The sensitizer peptide-fused endolysin Lys1S-L9P has highly effective bactericidal activity against various MDR Gram-negative bacteria. Lys1S-L9P displayed strong antibacterial action against MDR Gram-negative bacteria in the *G. mellonella in vivo* model, with no harmful effect. Sensitizer peptide-fused endolysin could be a useful biocontrol agent against MDR Gram-negative bacteria (Son et al., 2023).

## 5 Applications of endolysins

### 5.1 Endolysin is a disinfectant agent in the healthcare environment

*A. baumannii* is a leading contributor to nosocomial infections; it is becoming more and more linked to different epidemics and is a serious worry in intensive care units of hospitals across the globe (Almasaudi, 2018). Nosocomial infections resulting from multidrug-resistant bacteria are life-threatening (Choi et al., 2022). Secondly, the formation of bacterial biofilms on medical equipment is one of the biggest problems in the healthcare system (Gutiérrez et al., 2012). Bacteria trapped in biofilms are less sensitive because they grow more slowly and have restricted access to antibiotics and disinfectants (Davies, 2003). The bacteria-reduced susceptibility to antimicrobial agents within the biofilm that exhibited a more resistant phenotypic condition has also been linked to recurring infections (Lewis, 2007). Endolysins break down the bacterial cell wall peptidoglycan and could potentially help in controlling the spread of infections of MDR bacteria seen in hospitals (Choi et al., 2022). This revealed the endolysin potential as an environmentally friendly replacement for hospital-use harmful chemical disinfectants (Choi et al., 2022).

### 5.2 Role of endolysin in therapeutic protein delivery

**FIGURE 2:**
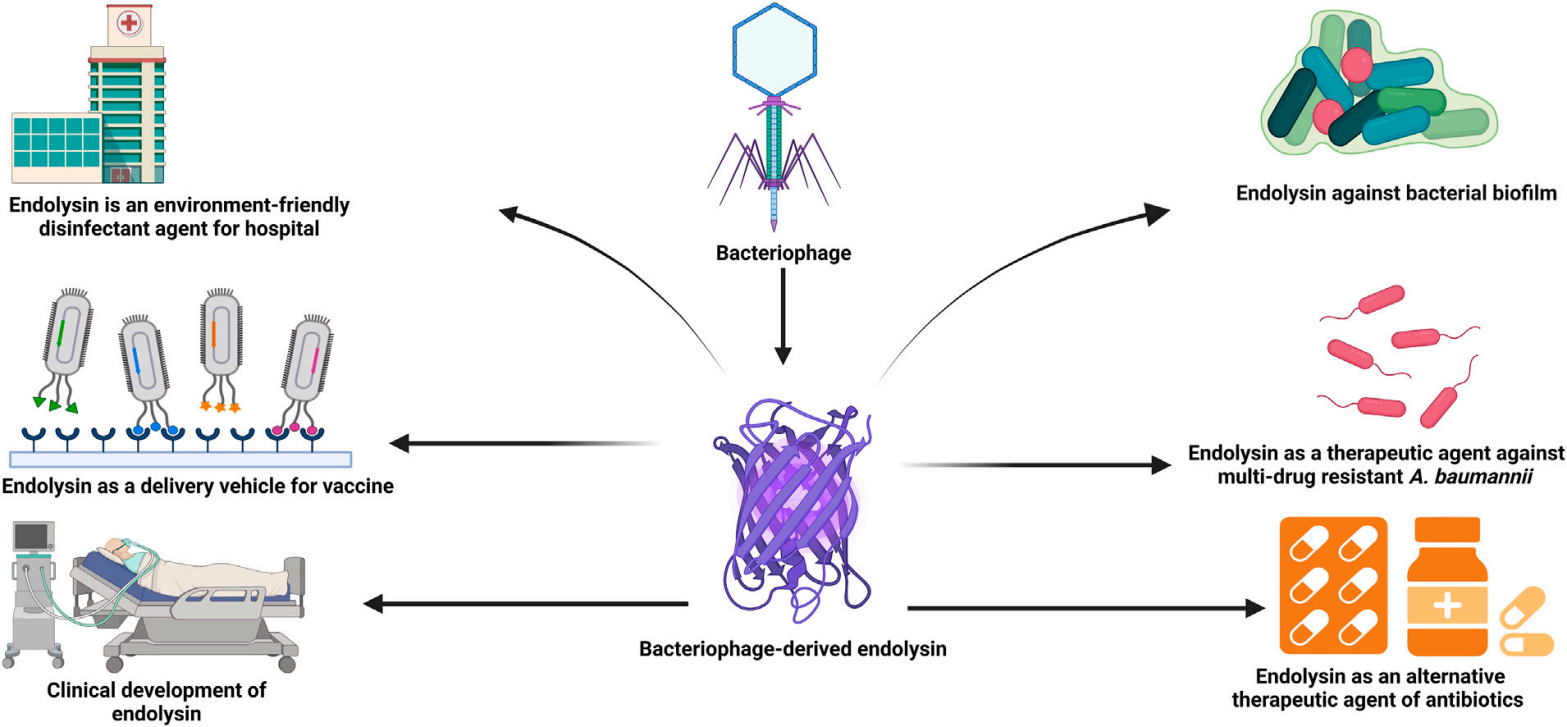
The figure illustrates the different applications of bacteriophage-derived endolysins.

Vaccination, which stimulates the immune system to enhance adaptive immunity through the injection of antigenic material, is one of the greatest public health achievements. Viral nanoparticles (VNPs) have been a focus of recent research as delivery vehicles for protein and peptide-based vaccinations (Rodríguez-Rubio et al., 2016b). Bacteriophage-derived nanoparticles have drawn the attention of researchers since foreign peptides or proteins can be expressed on the surface of phages as fusion proteins. As a result, phage-displayed peptides or proteins have been proven to be functionally and immunologically active, making them appropriate for vaccine development (Hamzeh-Mivehroud et al., 2013). Endolysin can also be employed as a binding agent for displaying heterologous proteins on the surfaces of bacteria in the hunt for live vaccination delivery systems. The endolysin CBDs, can be fused with various other proteins such as antigens to show them at the bacterial surface while retaining their structure and activity (Loessner et al., 2002). Surface display technology based on bacteriophage-derived endolysins has been developed using lactic acid bacteria (Hu et al., 2010; Ribelles et al., 2013). It has been shown that the external addition of the E7 antigen from human papillomavirus type-16 (HPV-16) when fixed on the surface of lactic acid bacteria by the CBD of *Lactobacillus* casei A2 phage endolysin, secure mice from an HPV-16 attack and its associated tumors. After being immunized *via* intranasal vaccination with lactic acid bacteria expressing E7 antigen, more than 60% of the mice remained tumor-free. Thus, the surface display of endolysins CBDs is a fascinating method for effectively and safely delivering therapeutic proteins (Ribelles et al., 2013). Figure 2, illustrates the applications of bacteriophage-derived endolysin.

## 6 Conclusion

Due to the lack of antibiotic development for Gram-negative bacterial infections, in a scenario in which no curative therapies are available, there is an urgent need to find innovative antibacterial drugs. While bacteriophage-encoded endolysin has been regarded as an alternative therapeutic agent against bacterial infections. For Gram-positive bacteria, there are various ongoing clinical trials in Phase I and Phase III, while endolysin development against Gram-negative bacterial infections is lagging. The presence of an outer membrane on the Gram-negative bacteria is a key barrier to its development because it prevents endolysin from accessing the underlying peptidoglycan substrates, limiting their potency. For this reason, increasing interest has been focused on permeabilizing endolysin that exerts lytic activity against Gram-negative bacteria throughout the outer membrane. Endolysin with synergistic effects with other antibacterial drugs against *A. baumannii* are summarized and described. To move clinical development forward, several challenges must be overcome, including safety concerns about the Gram-negative bacteria membrane-disrupting permeabilizers including EDTA, a lack of knowledge about immunogenicity and pharmacokinetics, lower potency against the stationary phase of Gram-negative bacteria and stability issues in serum and blood. While further *in vivo* research is needed to understand the safety and distribution profiles of endolysin after administration, protein engineering and formulation sciences are promising ways to improve the efficacy and stability of Gram-negative endolysin.
